# Selenium-integrated conjugated oligomer nanoparticles with high photothermal conversion efficiency for NIR-II imaging-guided cancer phototheranostics in vivo

**DOI:** 10.1186/s12951-023-02080-1

**Published:** 2023-09-04

**Authors:** Lele Yang, Yijian Gao, Jinchao Wei, Zehua Cheng, Sijia Wu, Liang Zou, Shengliang Li, Peng Li

**Affiliations:** 1 State Key Laboratory of Quality Research in Chinese Medicine, Institute of Chinese Medical Sciences, University of Macau, Macau, 999078 China; 2 College of Pharmaceutical Sciences, Soochow University, Suzhou, 215000 China; 3 School of Medicine, Chengdu University, Chengdu, 610106 China

**Keywords:** Photothermal therapy, Nanoparticles, NIR II-response, Fluorescence imaging, Cancer

## Abstract

Second near-infrared (NIR-II) fluorescence imaging in the range of 1000–1700 nm has great prospects for in vivo imaging and theranostics monitoring. At present, few NIR-II probes with theranostics properties have been developed, especially the high-performance organic theranostics material remains underexploited. Herein, we demonstrate a selenium (Se)-tailoring method to develop high-efficient NIR-II imaging-guided material for in vivo cancer phototheranostics. Via Se-tailoring strategy, conjugated oligomer TPSe-based nanoparticles (TPSe NPs) achieve bright NIR-II emission up to 1400 nm and exhibit a relatively high photothermal conversion efficiency of 60% with good stability. Moreover, the TPSe NPs demonstrate their photothermal ablation of cancer cells in vitro and tumor in vivo with the guidance of NIR-II imaging. It is worth noting that the TPSe NPs have good biocompatibility without obvious side effects. Thus, this work provides new insight into the development of NIR-II theranostics agents.

**Supplementary Information:**

The online version contains supplementary material available at 10.1186/s12951-023-02080-1.

## Introduction

Ranking as the leading cause of death nowadays, cancer remains an intractable disease with high and increasing mortality and morbidity worldwide [1, 2]. Approximately 10 million deaths of 18 million newly diagnosed patients with cancer are estimated globally in 2020, which advances the research on diagnostic strategies and new therapies [1, 3–5]. Traditional chemotherapy is widely used for cancer treatment, despite its limited therapeutic efficacy and frequent side effects. It directly inhibits tumor growth while potentially causing toxicity in normal tissues [6, 7]. To address these challenges, therapeutic strategies such as phototherapy, radiotherapy, and immunotherapy have exhibited good therapeutic outcomes and hold great promise for high-effect cancer treatments [3, 6, 8–13]. Among them, phototherapy that integrates therapy with diagnosis enabling in situ bioimaging and phototherapeutics has brought hope to cancer patients [14–20]. Despite the great potential of image-guided cancer therapy, new materials with great biological safety, high sensitivity, and high-resolution, real-time, and bright imaging are urgently needed.

Nanoparticles (NPs) possessing second near-infrared (NIR-II) fluorescence imaging (FLI) in the range of 1000–1700 nm have been extensively exploited as non-invasive diagnosis and therapy for various types of diseases [15, 21–25]. Compared with the first near-infrared (NIR-I) FLI (650–950 nm), the emergence of NIR-II FLI NPs materials with better signal-to-noise ratio, greater sensitivity, and deeper tissue penetration depth shows higher phototherapy efficacies [26–28]. To achieve long-wavelength NIR-II emission and bright NIR-II imaging, a series of NIR-II NPs including inorganic and organic materials have been constructed for phototherapy applications [29–34]. For instance, 1,2-distearoyl-sn-glycero-3-phosphoethanolamine-N-[methoxy(polyethylene glycol) micelles encapsulated t-BPITBT-TPE aggregates exhibited good imaging ability to cancer cell progression in xenografted zebrafish, as well as imaging 4T1 tumors in xenografted mice in vivo [35]. In another study, Dai and colleagues fabricated a novel molecular electron acceptor structured boron difluoride formazanate materials for NIR-II FLI-directed phototheranostics against tumors in animal bearing 4T1 tumor upon NIR light illumination [29]. Tang et al. developed an activatable fluorescence nanoprobe based on bio-erasable intermolecular donor-acceptor interaction to effectively evaluate inflammation tissues in live mice by NIR-II FLI of ClO^−^ [36]. Recently, side chain constructed semiconducting polymer NPs have been successfully designed and synthesized for NIR-II FLI and combating cancers both in vitro and in vivo [37]. NIR laser-responsive phototheranostic agents have been emerging as a promising strategy for imaging and oncotherapy owing to their unique advantages [20, 38–43]. It is an urgent demand to develop high-performance organic theranostics agents with good NIR-II FLI and high efficiency.

**Scheme 1:**
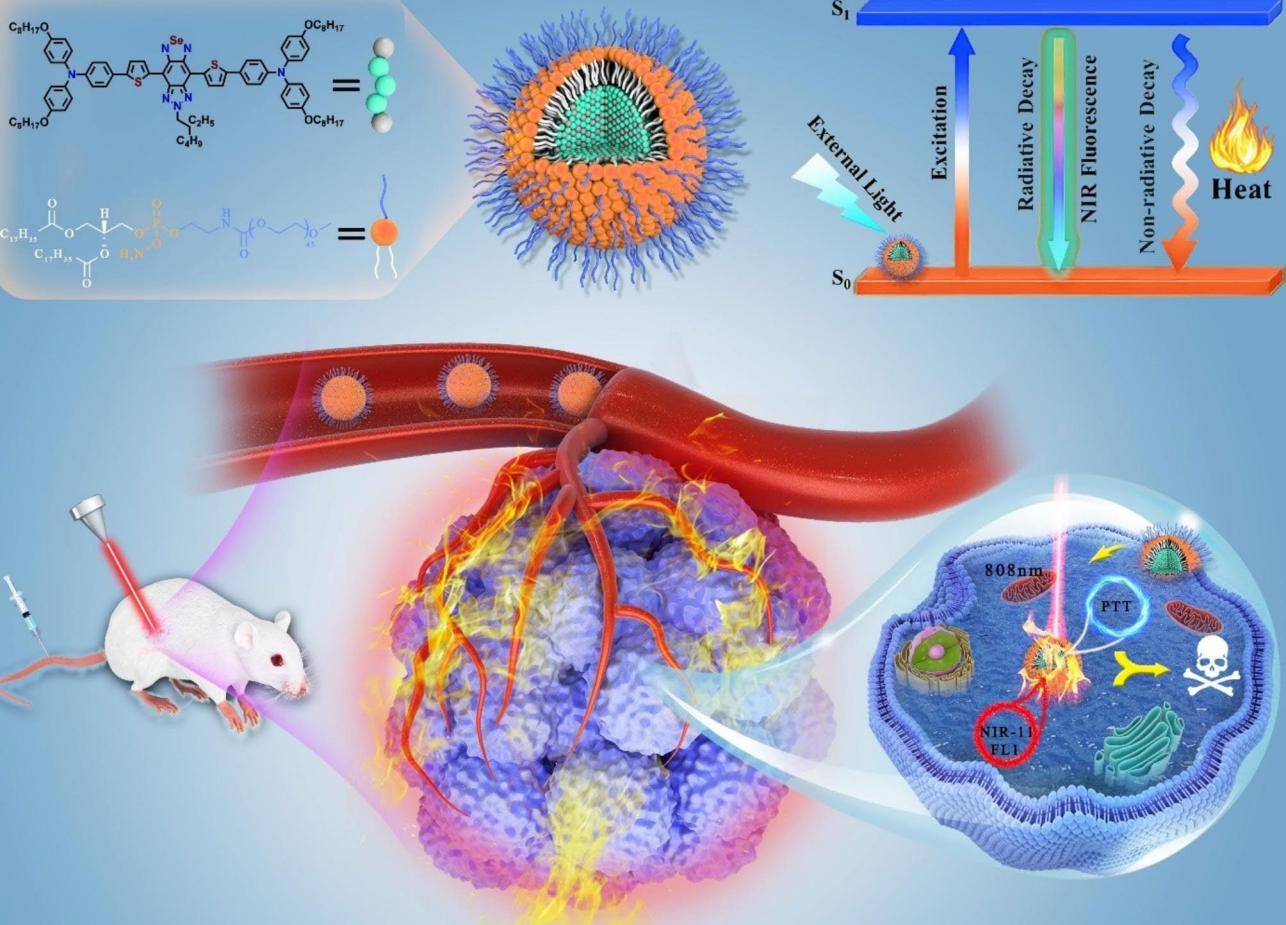
Schematic illustration of NIR-II-emissive conjugated oligomer nanoparticles for high-performance cancer phototheranostics upon NIR light irradiation

In this work, we designed conjugated oligomer TPSe NPs via selenium (Se)-tailoring strategy for high-efficient NIR-II imaging-guided cancer phototheranostics both in vivo and in vitro (Scheme 1). Under NIR laser irradiation, the organic theranostics TPSe NPs achieved satisfactory NIR FLI with NIR-II emission up to 1400 nm. With this whole-body NIR imaging, it provided high-resolution imaging of targeted tumor upon laser photoirradiation. Moreover, the obtained TPSe NPs had a higher photothermal conversion efficiency (PCE) of 60.29% with good stability than indocyanine green (ICG), which exhibited significant temperature changes and stable photothermal capability. Results proved that the TPSe NPs exhibited high-efficient tumor elimination capability in different cancer cell lines and a tumor mouse model. More importantly, histological examination of major tissues and biochemical indexes of blood revealed that little toxicity of the NPs against normal tissues was observed. Thus, this study introduces a novel strategy for NIR-II FLI-guided high-performance phototheranostics, which paves a different avenue for the designment of NIR-II theranostics agents.

## Experimental section

### Reagents and materials

DSPE-PEG2000 were obtained from Avanti Polar Lipids Inc. (Alabama, USA). ICG were obtained from Aladdin Chemical Co., Ltd (Shanghai, China). Fetal bovine serum (FBS), penicillin/streptomycin (PS), and trypsin were obtained from Thermo Fisher Scientific Inc. (Waltham, MA, USA). Other materials from Sigma-Aldrich (Saint Louis, MO, USA) were used without additional treatment.

### Characterization

High-resolution transmission electron microscopy image of TPSe NPs was acquired by a Talos F200X transmission electron microscope. Particle size was obtained by a Malvern Zetasizer Nano ZS 90. UV-VIS absorption spectra was obtained by employing a HACH DR6000 UV-vis spectrophotometer. Infrared thermal image was collected using a Fluke Ti480 Infrared Camera.

### Preparation of TPSe

Compound 4-(octyloxy)-N-(4-(octyloxy)phenyl)-N-(4-(5-(trimethylstannyl)thiophen − 2-yl)phenyl)aniline was synthesized according the reference [18], compounds 4,8-dibromo-6-(2-ethylhexyl)- [1, 2, 5]selenadiazolo[3,4-f]benzotriazole were obtained from WUXI Senior material (Wuxi, China).

Compound 4-(octyloxy)-N-(4-(octyloxy)phenyl)-N-(4-(5-(trimethylstannyl)thiophen-2-yl)phe nyl)aniline (149 mg, 0.2 mmol) and compound 4,8-dibromo-6-(2-ethylhexyl)- [1, 2, 5] selenadiazolo [3, 4-f] benzotriazole (99 mg, 0.2 mmol) were added into the two-neck reactor, and then 10 mL of degassed toluene was added with a 10 min of N_2_ flushing. After that, 20 mg Pd(PPh_3_)_4_ catalyst was added into the noted mixture with the protection of N_2_. Afterward, the solution was directly heated and held the temperature of 110 ℃ for 24 h under N_2_ environment. The resulted crude product was firstly extracted using CH_2_Cl_2_, and further purified by column chromatography to offer a dark green solid (126.6 mg, 51%). 1 H nuclear magnetic resonance (NMR) (400 MHz, Toluene-d8) δ 9.17 (d, J = 4.1 Hz, 2 H), 7.67 (d, J = 8.7 Hz, 4 H), 7.40 (d, J = 4.1 Hz, 2 H), 7.12 (d, J = 5.9 Hz, 4 H), 7.09 (t, J = 4.3 Hz, 8 H), 6.80 (d, J = 8.9 Hz, 8 H), 4.47 (d, J = 6.5 Hz, 2 H), 3.67 (t, J = 6.4 Hz, 8 H), 1.69–1.61 (m, 8 H), 1.39–1.21 (m, 49 H), 0.94–0.89 (m, 18 H); 13 C NMR (101 MHz, Toluene-d8) δ 155.93, 148.69, 143.33, 140.73, 137.51, 136.80, 132.67, 128.73, 128.50, 128.26, 127.83, 127.59, 127.35, 126.75, 126.53, 125.00, 124.76, 124.52, 120.81, 115.36, 67.84, 31.97, 29.55, 29.51, 29.45, 26.24, 23.06, 22.80, 14.01, 10.41ppm; high-resolution mass spectrometry (HRMS): m/z: [M] + calcd for C90H113N7O4S2Se, 1499.75; found, 1499.74556.

### Preparation of TPSe NPs

TPSe molecule (0.5 mg) and DSPE-PEG2000 (0.5 mg) were weighted and dissolved in THF (1 mL). The solution was mixed thoroughly under sonication, and then injected into deionized water (9 mL) to obtain TPSe NPs under vigorous stirring. The obtained solution was successively purified with 0.22 μm filtration and concentrated by using centrifugal filter unit with a size of 100 kDa. The final NPs dispersion was kept at 4 ℃ until further experiments.

### Examination of photothermal performance

The examination of PCE was performed as described in our previous report [44]. Typically, a 0.8 mL amount of TPSe NPs solutions with increased concentration from 0 to 40 µg mL^− 1^ was illuminated at 1 W cm^− 2^ for 6 min using an 808 nm light. After steady state temperature, excitation light was turned off until the temperature returns to the initial state. Thermal images were acquired and analyzed by a Fluke Ti480 Infrared Camera. The calculation of η was briefly described as following Eq. (1):

1 $$\eta =\frac{hS\left({T}_{max}-{T}_{surr}\right)-{Q}_{DI}}{I(1-{10}^{-{A}_{\lambda }})}$$

where *η* stands for photothermal conversion efficiency, *h* is the heat transfer coefficient, *S* is the surface area of cuvette exposed to laser irradiation. *T*_*max*_ and *T*_*surr*_ stand for the temperature reached at thermal equilibrium state and the surrounding temperature, respectively. *Q*_*DI*_ stands for the energy input from deionized water in the detected solution. *I* is the power density of 808 nm laser, A_*λ*_ is the absorbance intensity of TPSe NPs solution upon irradiation. *hS* could be calculated according to Eq. (2):

2 $${\tau }_{S}=\frac{{m}_{D}{C}_{D}}{hS}$$

where, m_*D*_ and C_*D*_ stand for the mass (0.8 g) and heat capacity (4.2 J g^− 1^) of DI water, respectively. τ_s_ stands for the time constant for heat transfer, which could be calculated according to Eq. (3):

3 $${t=-\tau }_{S}\text{ln}\left(\theta \right)={-\tau }_{S}\text{ln}\left(\frac{{T}_{t}-{T}_{Surr}}{{T}_{Max}-{T}_{Surr}}\right)$$

where, *t* stands for the cooling time points after laser irradiation for 10 min, *T*_*t*_ stands for the recorded temperature of TPSe NPs solution during the cooling stage.

### In vitro photostability test

To evaluate the photostability of TPSe NPs, NPs of 40 µg mL^− 1^ (0.8 mL) were illuminated at 1 W cm^− 2^ under 808 nm light for 1 h, while ICG of 30 µg mL^− 1^ was selected as the control sample. Furthermore, photothermal conversion capability of TPSe NPs solution was investigated under ten laser on/off irradiation cycles, and ICG was selected as the control group.

TPSe NPs solution was irradiated by 808 nm laser for 10 min, then the size, zeta potential, and absorption spectra of TPSe NPs before and after irradiation were measured to evaluate the photostability of TPSe NPs.

### In vitro ROS generation

Firstly, DCFH-DA was hydrolyzed to DCFH by 0.01 N NaOH, and 1 × PBS was used to terminate the hydrolysis reaction. DCFH solution (5 µmoL) was added with or without 30 µg mL^− 1^ TPSe NPs. The fluorescence intensity of the DCFH was detected every 1 min under the 808 nm laser irradiation.

### In vitro cytotoxicity of TPSe NPs

The cytotoxic effects of NPs on various cancer cell lines (HCT116, A549, 4T1, MCG-803, HepG2) were evaluated by CCK-8 assay (Beyotime Biotech Inc, China). Typically, the HCT116 human colorectal cancer cells were suspended in 1640 medium supplemented with 10% FBS and 1% PS in an incubator at 37 °C, 5% CO_2_ atmosphere. Cell (5 × 10^3^ cells/well) was cultured in a 96-well plate for 24 h and then challenged with NPs of various concentrations (6.25-30 µg mL^− 1^) for 4 h. After 5 min irradiation with or without an 808 nm laser (0.5 W cm^− 2^), cells were cultured for a further 24 h. Afterward, cell viability was determined using CCK-8 kits to evaluate the cytotoxicity of NPs.

### Live-dead cell staining

In total, cells (2 × 10^5^) were plated in confocal dishes. After attachment, the cell was treated with PBS or NPs (30 µg mL^− 1^) and then cultured for 4 h. Afterward, cells were illuminated with or without an 808 nm light for 5 min (0.5 W cm^− 2^). Cells were washed with PBS three times, followed by incubation with Calcein-AM/Propidium Iodide (Beyotime Biotechnology) in the dark for 30 min. After washing twice using PBS, fluorescence was monitored through a DMi8 Platform Live cell microscope (Leica Microsystems, Germany).

### Tumor mouse model

All mice procedures were conducted according to the Animal Management and Ethics Committee of Soochow University. BALB/c nude mice of 4∼6 weeks were provided by Beijing Vital River Laboratory and housed for 1 week. Afterward, the HCT116 cell was injected in the right upper limb of nude mice (2 × 10^7^ cells mL^− 1^, 100 µL), when the cell growth density is about 80%. Once tumors reached ~ 100 mm^3^, mice were subsequently used for NIR FLI and antitumor tests.

### In vivo NIR-II FLI

In vivo NIR-II fluorescence image was acquired using an NIR-II imaging equipment with indium-gallium-arsenide (InGaAs) array. The equipment parameters were fixed and used for the following in vivo imaging (excitation wavelength 808 nm; power density 60 mW cm^− 2^; long pass filter 1050 nm; exposure time 300 ms). The BALB/c nude mice were injected with TPSe NPs (100 µL, 0.5 mg mL^− 1^) intravenously (n = 3) when the tumor volume is about 100 mm^3^. And they were kept anaesthetized via a nose cone during injection and imaging acquisition, which delivered 2 L min^− 1^ oxygen flow mixed with 3% isoflurane. NIR-II images of different tissues and tumors of the animals were acquired after 36 h postinjection. The calculation of fluorescence signal intensities of images obtained was quantitatively performed using Image J. The biodistribution of TPSe NPs was analyzed based on the fluorescent intensity of excised organs.

### In vivo antitumor activity

To investigate anti-tumor activity of NPs in vivo, the established tumor models were randomized into different groups (n = 4). The mice in experimental group were irradiated upon 808 nm light for 5 min with 0.5 W cm^− 2^ at 24 h after intravenous administration of either 100 µL of 0.5 mg mL^− 1^ NPs [TPSe NPs (0.5 mg mL^− 1^) + L] or with 100 µL of 1 mg mL^− 1^ NPs [TPSe NPs (1.0 mg mL^− 1^) + L]. Mice administrated with PBS, mice administrated with PBS and illuminated with laser (PBS + L), and mice injected with 0.5 mg mL^− 1^ of NPs [TPSe NPs (0.5 mg mL^− 1^)] were used as the control. The tumor temperatures were observed and recorded using a thermal camera. Tumor growth and mouse weight were monitored every 2 days during the whole experiment.

### Biosafety assessment

On day 12, the tumor tissues were taken for weight measure, and several other tissues, including heart, liver, spleen, lung, kidney were obtained for H&E assay. The hematologic and biochemistry analysis of blood samples were performed using an automated hematology analyzer.

## Results and discussion

### Design, synthesis, and characterization of TPSe

The conjugated oligomer TPSe was designed with a typical acceptor-donor-acceptor (A-D-A) construction and Se-tailoring to achieve good NIR-response performance. For molecular synthesis, the electron-donating triphenylamine (TPA, A fragment) and electron-withdrawing thiadiazolobenzotriazole (TBZ, D fragment) were conjugated *via* Stille coupling reaction (Additional file 1: Figure S1). The TPA donor was attached to both sides of TBZ to obtain a lower band gap construction of “A-D-A”, while the thiophene in the middle of TBZ was replaced with Se to enhance the heavy-atom effect [28]. The precise molecular structure was verified by NMR and HRMS (Additional file 1: Figures S2-S4). UV-VIS-NIR absorption spectrum of TPSe were tested in THF solvent, and shows typical maximum absorbance at 830 nm (Additional file 1: Figure S5). Density functional theory (DFT) investigation indicated that the highest occupied molecular orbital (HOMO, -4.36 eV) and the lowest unoccupied molecular orbital (LUMO, -3.08 eV) of TPSe show good distribution in the D and A units, and their energy gap further matches well to absorption spectrum (Additional file 1: Figure S6).

### Preparation and characterization of NPs

**Fig. 1:**
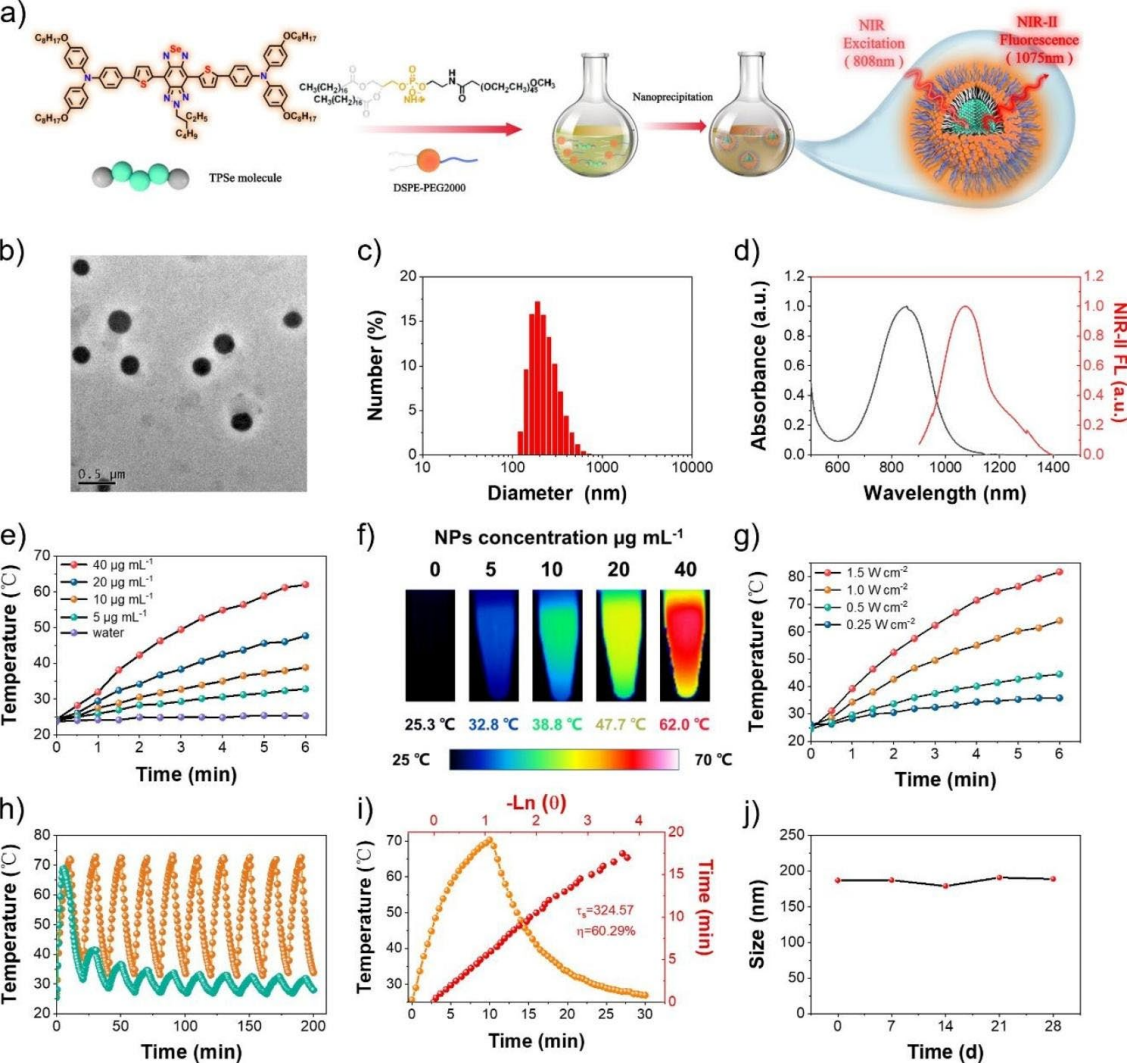
**(a)** Schematic illustration of synthesis and preparation of TPSe NPs. **(b)** TEM image of TPSe NPs. **(c)** Dynamic light scattering of TPSe NPs. **(d)** Absorption spectral and NIR-II fluorescence of TPSe NPs (DI water). **(e)** Changes in photothermal temperature of water and TPSe aqueous solutions of varied concentrations. **(f)** Infrared images of TPSe NPs solutions in the sixth minute upon laser illumination. **(g)** Photothermal heating curves of TPSe aqueous solutions (40 µg mL^− 1^) upon laser irradiation with various powers of 808 nm light. **(h)** Temperature profiles of TPSe NPs and ICG irradiated at 1 W cm^− 2^ by 808 nm light for 10 laser on/off cycles. **(i)** Temperature profile of TPSe NPs (40 µg mL^− 1^) under 808 nm laser irradiation for 10 min at 1 W cm^− 2^ with subsequent natural cooling process after turning off the laser (yellow curve) and the linear time data versus -Ln (θ) obtained from the cooling process (red curve). **(j)** Stability test of TPSe NPs kept at 4 °C for 28 days

To evaluate the potential in photoactivable biological applications, water-dispersible TPSe nanoparticles (TPSe NPs) were prepared according to nanoprecipitation strategy (Fig. 1a). It has been widely reported that the presence of PEG shell could decrease the interactions between the biological environment and the PEG-functionalized nanomaterials. The circulation of PEG-modified materials was found to be longer as compared to the nanomaterials without surface functionalization [45–47]. As a classical surface functionalization approach, DSPE-PEG2000 modification has been widely applied in the decoration of various nanomaterials, enabling their stability and prolong blood circulation [48]. According to the calculation method, the encapsulation efficiency of TPSe was 53.5% with a drug loading rate of 14.4% (Additional file 1: Materials and methods). Thus, the very facile and highly repeatable encapsulation by amphiphilic copolymer DSPE-PEG2000 was successfully performed to protect TPSe NPs from absorption at nonspecific sites. As shown in Fig. 1b and c, TPSe NPs were obtained with uniform spherical morphology and a diameter of about 200 nm, as indicated by the transmission electron microscopy (TEM) and dynamic light scattering (DLS). As previously reported, materials with the size between 10 and 1000 nm usually exhibit enhanced permeability and retention (EPR) effect [49], thus it is possible for TPSe NPs to passive-target accumulate in the tumor for further cancer therapy. Figure 1d and S5 exhibits the absorption spectral of TPSe together with red-shift due to the aggregation from molecular (830 nm) to nanoparticle (870 nm), which largely enhances the NIR absorption efficiency and then contributes to the excellent NIR-II fluorescent emission (1075 nm). The produced red shifts of absorption spectrum could be a result of J-aggregate state of TPSe NPs upon nanoparticle construction, which has been reported in various materials, such as oligomer nanoparticles and ICG [50]. The strengthened efficiency of NIR absorption is highly beneficial for high-performance cancer phototheranostics upon NIR light irradiation [50]. Due to the maximum absorption at 870 nm is more closed to the excitation wavelength 808 nm commonly used in photothermal therapy, TPSe NPs exhibited significant temperature changes under laser irradiation. The concentration-dependent changes in the temperature were exploited. With the concentration of TPSe NPs increased from 0 to 40 µg mL^− 1^, significant temperature elevation was observed apparently (Fig. 1e). Moreover, the NIR photothermic conversion capability of NPs solutions was then evaluated by using an infrared camera. The temperature of TPSe NPs solutions (40 µg mL^− 1^) was increased rapidly to 62 ℃ upon 808 nm light (1.0 W cm^− 2^) irradiation within six minutes (Fig. 1e). Correspondingly, infrared images were obtained and shown in Fig. 1f. The power density-dependent manner of TPSe NPs induced temperature rise was also investigated (Fig. 1g). And then, the photostability of TPSe NPs was studied by laser irradiation for 1 h at 1.0 W cm^− 2^ using an FDA-approved clinical fluorescent medicine ICG as the control. It is encouraging that almost no difference in the absorption spectra, color and size distribution of TPSe NPs was observed before and after laser irradiation (Additional file 1: Figure S7-S9). In contrast, a severe photobleached effect was observed in ICG solution, resulting in apparent changes in UV-absorption spectra. The zeta potential of TPSe NPs before and after irradiation were examined to further prove the photothermal stability of TPSe NPs. As shown in Additional file 1: Figure S10, there is no obvious change between the zeta potential of TPSe NPs before and after irradiation, indicating relatively stable properties of TPSe NPs. Most importantly, we further evaluated the photothermal performance of TPSe NPs upon 10 laser heating cycles. As shown in Fig. 1h, the prepared TPSe NPs displayed the outstanding and stable photothermal capability, whereas ICG showed diminishing trend during successive laser heating cycles. Finally, the TPSe NPs with PCE (η) of 60.29% were prepared and determined (Fig. 1i). Its stability was tested by DLS method over a 28-day period of storage, which displays little change in nanoparticle size (Fig. 1j), further confirming its photothermal stability. The photodynamic effect of TPSe NPs under laser irradiation was measured by fluorescent probe DCFH-DA (2’,7’-Dichlorodihydrofluorescein diacetate). As shown in Additional file 1: Figure S11, TPSe NPs could produce negligible ROS under 808 nm laser irradiation (1.0 W cm^− 2^). Therefore, the killing effect of TPSe NPs on tumor cells mainly depends on the photothermal effect.

### In vitro photothermal therapy

**Fig. 2:**
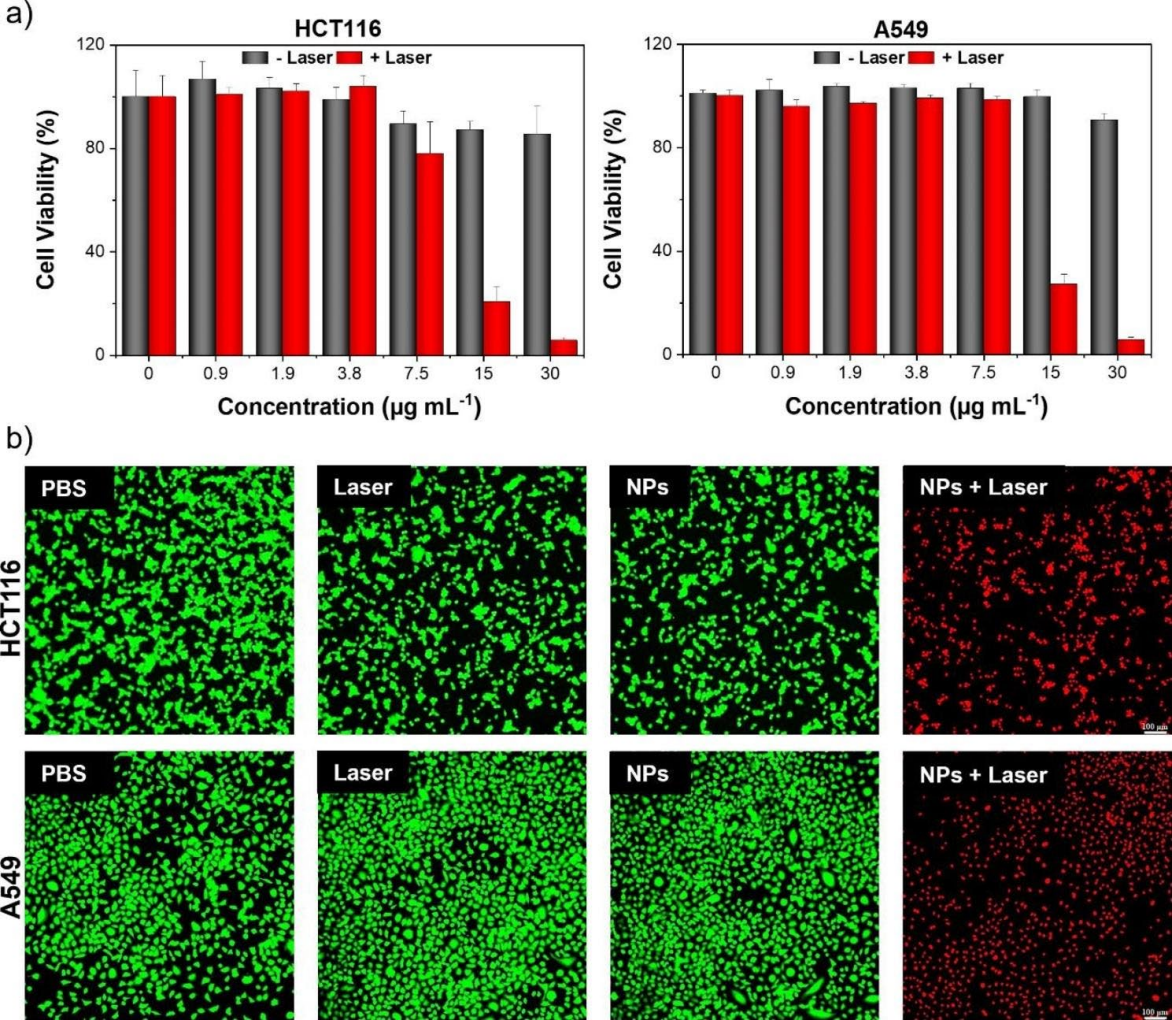
**(a)** In vitro survival rate of HCT116 cells and A549 cells after challenge with increasing concentration of TPSe NPs with or without laser illumination for 5 min at 808 nm (0.5 W cm^− 2^). **(b)** Fluorescence imaging of HCT116 cells (top) and A549 cells (down) co-stained with Propidium Iodide (dead cells emitting red fluorescence) and Calcein AM (live cells emitting green fluorescence)

To study the potential photothermal efficiency of TPSe NPs, in vitro cytotoxic effect of TPSe NPs on cancer cells was determined using CCK-8 kits. As illustrated in Fig. 2a, treatment of HCT116 colorectal carcinoma cell line and A549 lung cancer cell line with TPSe NPs for 24 h displayed minimal cytotoxicity even after incubation with TPSe NPs at a high concentration, suggesting its good in vitro biocompatibility. By contrast, the cell viability of TPSe NPs-treated cancer cells was significantly degraded after 5 min laser irradiation, with severe cell death (about 90%) at a high concentration (30 µg mL^− 1^). This excellent tumoricidal efficacy of TPSe NPs was also confirmed on other three cancer cell lines, i.e., 4T1, MGC-803, HepG2 (Additional file 1: Figure S12). Given the potential photothermal toxicity of TPSe NPs in cancer cells, therapeutic effect of TPSe NPs was further confirmed by visible live/dead cell staining assay. The cells were co-stained with Calcein-AM (live cells emitting green color) and Propidium Iodide probes (dead cells emitting red color). As illustrated in Fig. 2b, HCT116 and A549 cell challenged with PBS, laser irradiation, or TPSe NPs without irradiation exhibited green fluorescence, which suggested minimal impacts on cell viability. Upon laser illumination with 808 nm for 5 min (NPs + Laser), almost no green fluorescence was observed in TPSe NPs-treated cells, confirming its efficient photothermal ablation of cancer cells. These results collectively suggest that TPSe NPs could serve as a promising phototherapy material for photothermal elimination of tumor.

### In vivo NIR-II FLI, antitumor effect, and biosafety of TPSe NPs

**Fig. 3:**
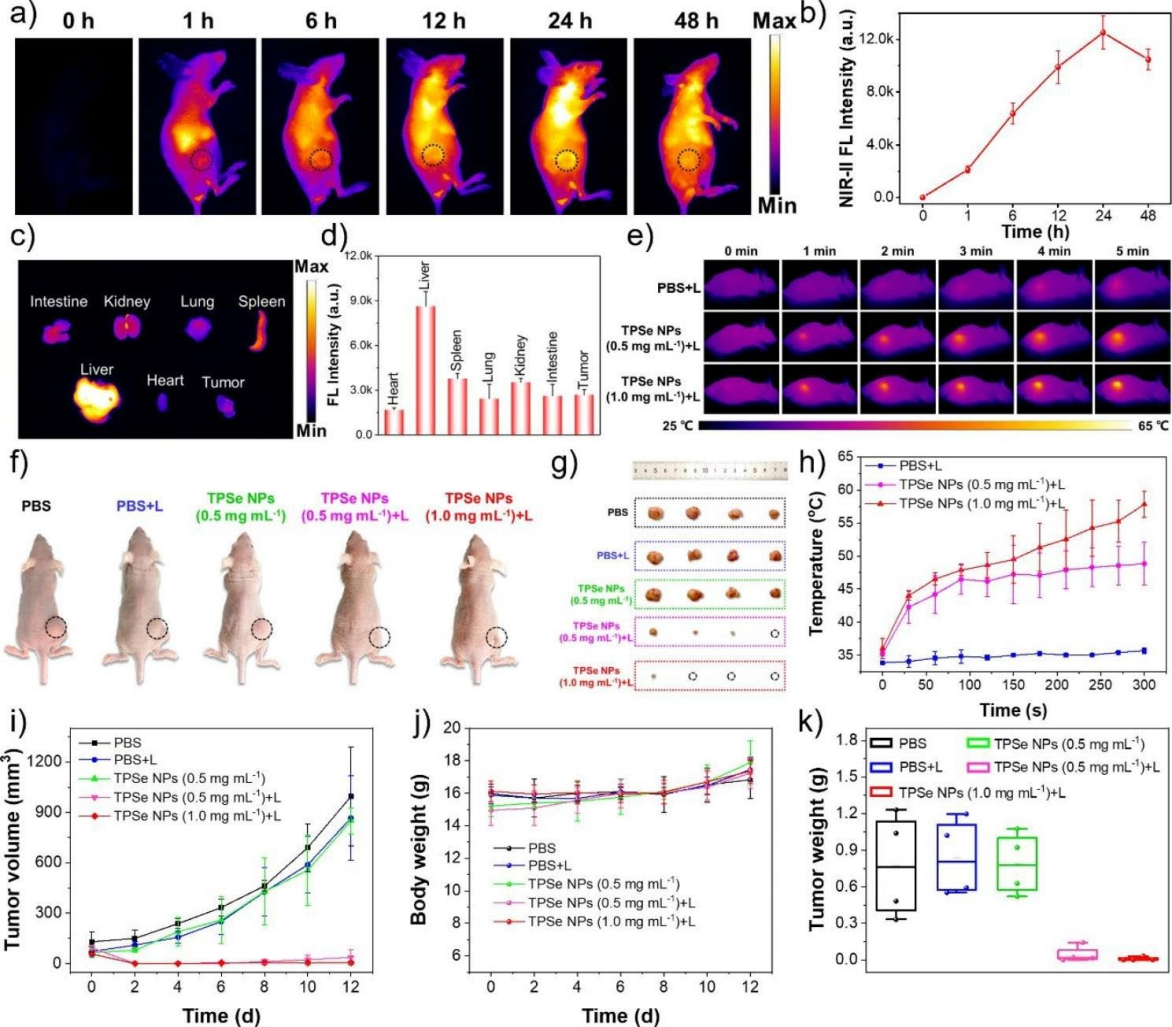
**(a)** In vivo fluorescence imaging of HCT116 tumor-bearing mice at 0, 1, 6, 12, 24 and 48 h after intravenous administration of TPSe NPs. **(b)** Fluorescence intensity from **(a)** plotted as a function of time post-injection. **(c)** Fluorescence image and **(d)** fluorescence intensity of tumor and other major organs, including heart, liver, spleen, lung, kidneys and intestine after 24 h post-injection of TPSe NPs. **(e)** Thermal image of the mice in PBS + Laser (L) group and TPSe NPs (0.5 mg mL^− 1^ or 1.0 mg mL^− 1^) + L group upon irradiation and **(h)** temperature profiles of tumor regions upon irradiation using 808 nm laser. **(f)** Representative photograph of mice and **(g)** tumors in different groups after 12 days treatment. **(i)** Tumor volume changes of mice between 12 days. **(j)** Weight of mice in different groups between 12 days. **(k)** Tumor weight of mice at 12 days

In order to verify the in vivo phototherapy application of TPSe NPs, their NIR-II FLI, antitumor effect, biocompatibility and biosafety were systematically investigated. Encouraged by the superior photothermal ablation efficacy of TPSe NPs in vitro, human colorectal adenocarcinomas-xenografted mice were used to evaluate the in vivo photothermic performance of TPSe NPs. Briefly, the tumor-xenograft models were employed by subcutaneous injection of HCT-116 cells into nude mice. NIR-II FLI of tumors provides efficacious guidance upon laser illumination (808 nm) after intravenous administration with TPSe NPs before photothermal therapy. As shown in Fig. 3a, the NIR-II FLI of mice after intravenous administration with TPSe NPs of 0.5 mg mL^− 1^ at different time point were acquired with a filter of 1050 nm. And the fluorescence intensity of tumor (black line circle area) displayed a growing trend gradually from 0 to 24 h, then decreased significantly at 48 h because of the effective metabolic effect of TPSe NPs in mice. The maximum fluorescence intensity at 24 h post-injection suggested the good tumor-targeting performance of TPSe NPs and time requirement as fluorescence manuduction for photothermal therapy (Fig. 3a, b). Biodistribution after 24 h injection demonstrated the distribution of TPSe NPs in major organs in vivo and its effective accumulation in tumor (Fig. 3c, d). The ex vivo NIR-II FLI of major organs was determined at 24 and 48 h post-injection. As shown in Additional file 1: Figure S13, the NIR-II FL intensity at 48 h post-injection was significantly lower than that of 24 h post-injection, which indicates that the TPSe NPs could be metabolized from the circulatory system after 48 h. It is clear that the majority of the TPSe NPs were taken into the tissues consisting of abundant reticuloendothelial system, particularly the liver, the spleen and the kidney, leading to the clearance of the TPSe NPs from the circulatory system after injection.

Then the HCT116 human colorectal adenocarcinomas-xenografted mice were randomly assigned to five different groups: PBS, PBS + Laser (L), TPSe NPs (0.5 mg mL^− 1^), TPSe NPs (0.5 mg mL^− 1^) + L and TPSe NPs (1.0 mg mL^− 1^) + L. PBS and TPSe NPs (0.5 mg mL^− 1^ or 1.0 mg mL^− 1^, 100 µL) were intravenously administrated into mice bearing HCT116 tumor-xenograft model to assess photothermal therapy in vivo when tumor volume reached ~ 100 mm^3^. After 5 min of laser illumination at 0.5 W cm^− 2^ (808 nm), change in the temperature of mice tumor site in the TPSe NPs + L groups increased significantly within 5 min, affording adequate hyperthermia for tumor ablation. On the contrary, that of PBS + L group was almost negligible, indicating little photothermal effect of the employed laser illumination (808 nm, 0.5 W cm^− 2^). Additionally, the temperature change has an extremely high correlation with the concentration of TPSe NPs, and the temperature rose to 48.8 ℃ in TPSe NPs (0.5 mg mL^− 1^) + L group but 57.9 ℃ in TPSe NPs (1.0 mg mL^− 1^) + L group (Fig. 3e, h).

**Fig. 4:**
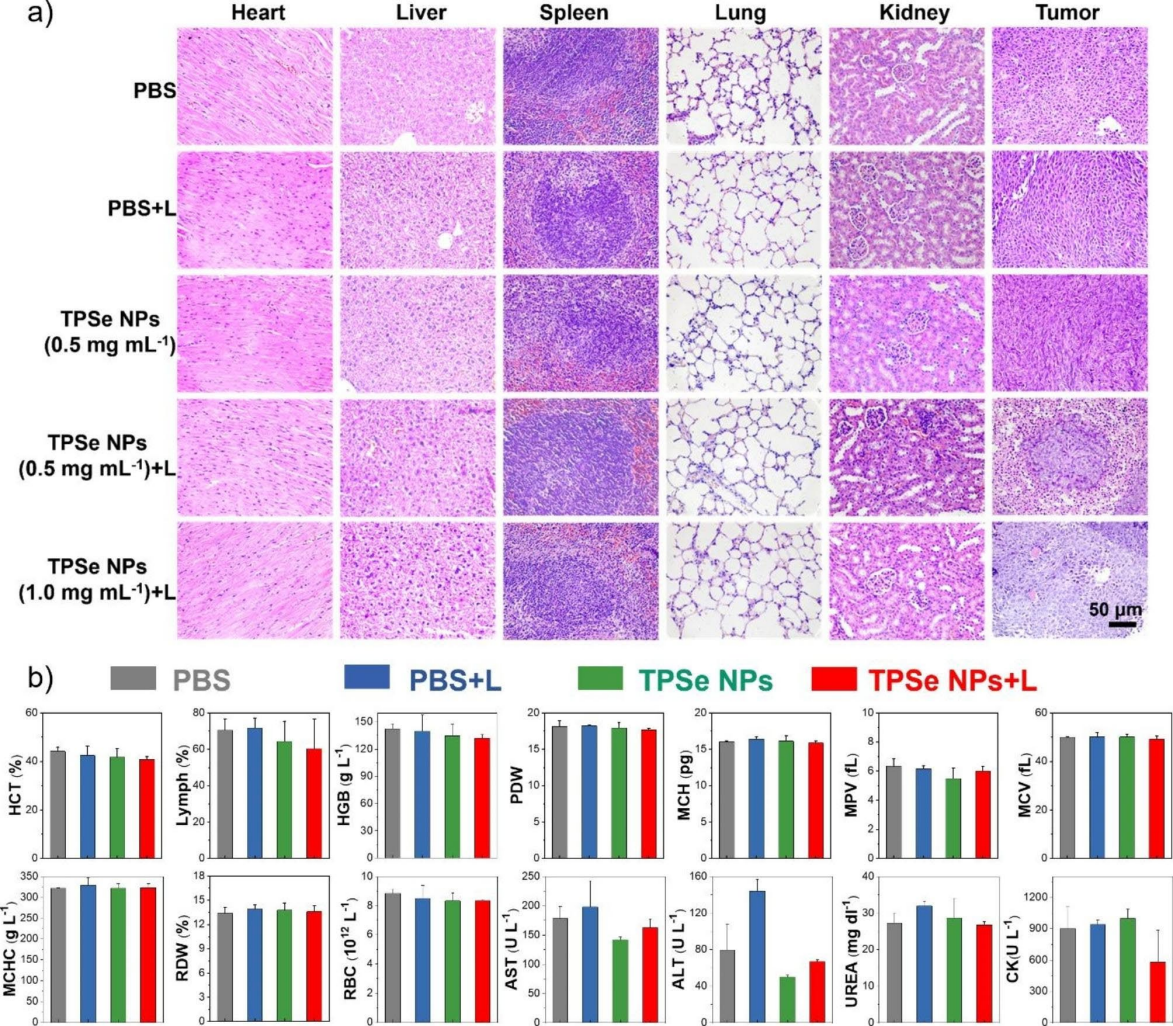
**(a)** Images of H&E-stained heart, liver, spleen, lung, kidney and tumor of mice. **(b)** Blood routine and biochemical indexes of mice after different treatments

After above treatment, the tumor volume and body weight of all groups were recorded every two days over a period of 12 days. After 12 days of various treatments, photographs and tumor tissues of the mice were taken. The representative photos of tumor-bearing mice in each group and tumors are displayed in Fig. 3f, g. The tumors of mice in PBS, PBS + L, and TPSe NPs (0.5 mg mL^− 1^) groups all increased significantly, while the tumors of mice in TPSe NPs (0.5 mg mL^− 1^) + L group were significantly smaller than other groups. More importantly, the tumors in the TPSe NPs (1.0 mg mL^− 1^) + L group almost completely disappeared, which fully proves good photothermal anti-tumor effect of TPSe NPs. After 12 days, the tumor volume (Fig. 3i) and tumor mass (Fig. 3k) of the TPSe NPs + L groups were all significantly reduced compared with the other groups, which further verified the above statement. After 12 days of phototherapy, the tumor sites were stained with hematoxylin and eosin (H&E) to confirm the efficacy of the treatment. According to Fig. 4a, the tumors of mice in PBS, PBS + L, and TPSe NPs group showed a high number of intact nuclei. The tumors treated with TPSe NPs (0.5 mg mL^− 1^) + L or TPSe NPs (1.0 mg mL^− 1^) + L displayed little proliferating cell nucleus. In conclusion, TPSe NPs as a phototherapeutic agent can integrate imaging and therapy, providing potentiality for the application of nanoparticles in cancer therapy.

During treatment, collected result of mice weight in different groups (Fig. 3j) demonstrated that the mice weight did not show significant differences and gradually increased, indicating few side effects of TPSe NPs in vivo. The proved biosafety of TPSe NPs provides a certain guarantee for its further application in anti-tumor therapy. To further confirm the in vivo biological security of TPSe NPs, examination of H&E staining of major organs and hematology markers were performed after the indicated therapy. The images of H&E-stained tissues of mice including heart, liver, spleen, lung, and kidney in each group after treatment are shown in Fig. 4a. The ignorable difference of the main organs between each group certifies that the TPSe NPs didn’t cause any damage to the organs. In addition, blood routine examination and blood biochemical parameters such as HCT, Lymph, AST, and ALT were also collected to verify the biocompatibility of TPSe NPs. Figure 4b illustrates the blood indexes of the different groups were all within the normal range, and the liver and kidney functions were not damaged by TPSe NPs. Taken together, it revealed the satisfactory anti-tumor efficacy and biosafety of TPSe NPs in the current treatments for mice. Next, we compared the property of the constructed TPSe NPs with reported materials from recently papers in terms of PCE, laser wavelength, laser irradiation time, maximum temperature etc. to gain further insight on its advantages and limitations (Additional file 1: Table S1). Among the reported inorganic nanomaterials and organic nanomaterials for bioimaging and phototheranostics, organic small molecules with great potential of clinical translation have attracted increasing interest because of their superior efficacy of photothermal therapy and high biocompatibility [49, 51]. Moreover, organic materials with NIR-II FLI and high PCE remain to be explored [52]. The designed TPSe NPs will enrich the toolbox for preparing high-performance organic theranostics agents with good NIR-II FLI and high efficacy.

## Conclusion

In summary, the preparation of TPSe NPs with NIR-II emission up to 1400 nm under excitation at 808 nm have been successfully performed using facile nanoprecipitation approach. By taking advantage of the aggregation strategy, the constructed TPSe NPs exhibit a strong absorption peak at 870 nm and achieve considerably high PCE of 60.29%. The whole-body NIR-II fluorescence bioimaging of TPSe NPs allows them to image accurate position of tumors, which displays high tumor accumulation capacity. The in vitro and in vivo experiments demonstrate that TPSe NPs has strong photothermal efficacy and high efficiency in tumor clearance. Furthermore, the biosafety analysis verified that TPSe NPs have good biocompatibility without obvious biological toxicity in vitro and in vivo. Overall, the facile strategy we proposed provides a hopeful strategy for developing effective NIR-II phototherapy agents. However, this study retains the laser power of 0.5 W cm^− 2^ for phototherapy tests. Next, the standard requirement of laser exposure (0.33 W cm^− 2^, American National Standard Institute) should be further employed for all in vitro and in vivo therapy applications. This work is a preliminary study and remains further clinical translation study to perform.

### Electronic supplementary material

Below is the link to the electronic supplementary material.

**Additional file 1** (1) Materials and methods 1.1. The encapsulating efficiency of the NPs. (2) Results Figure S1: Synthetic route of TPSe; Figure S2: 1 H NMR spectra of TPSe; Figure S3: 13 C NMR spectra of TPSe; Figure S4: High-resolution mass spectrometry of TPSe; Figure S5: UV-VIS-NIR absorption spectrum of TPSe; Figure S6: HOMO and LUMO of TPSe; Figure S7: Absorption spectra of TPSe before and after laser irradiation; Figure S8: Absorption spectra of ICG before and after laser irradiation; Figure S9: Size distribution of TPSe before and after laser irradiation; Figure S10: Zeta potential of TPSe NPs before and after irradiation; Figure S11: Detection of in vitro ROS generation; Figure S12: Toxicity of TPSe NPs in 4T1, MGC-803, HepG2 cell; Figure S13: Fluorescence image of major organs after injection of TPSe NPs; Table S1: The comparison of TPSe NPs with reported nanomaterials.

## References

[1] Bray F, Ferlay J, Soerjomataram I, Siegel RL, Torre LA, Jemal A (2018). Global cancer statistics 2018: GLOBOCAN estimates of incidence and mortality worldwide for 36 cancers in 185 countries. CA Cancer J Clin.

[2] Ferlay J, Colombet M, Soerjomataram I, Parkin DM, Pineros M, Znaor A, Bray F (2021). Cancer statistics for the year 2020: an overview. Int J Cancer.

[3] Chen H, Wan Y, Cui X, Li S, Lee CS (2021). Recent advances in Hypoxia-Overcoming Strategy of Aggregation-Induced Emission Photosensitizers for efficient photodynamic therapy. Adv Healthc Mater.

[4] Sung H, Ferlay J, Siegel RL, Laversanne M, Soerjomataram I, Jemal A, Bray F (2021). Global cancer statistics 2020: GLOBOCAN estimates of incidence and mortality worldwide for 36 cancers in 185 countries. Ca-a Cancer Journal for Clinicians.

[5] Wild CP (2019). The global cancer burden: necessity is the mother of prevention. Nat Rev Cancer.

[6] Zhao H, Guo YD, Yuan A, Xia SP, Gao ZQ, Huang YM, Lv FT, Liu LB, Wang S (2022). Nature-inspired nanothylakoids for multimodal cancer therapeutics. Sci China-Materials.

[7] Younis MR, Wang C, An RB, Wang SJ, Younis MA, Li ZQ, Wang Y, Ihsan A, Ye DJ, Xia XH (2019). Low power single laser activated Synergistic Cancer Phototherapy using Photosensitizer Functionalized Dual Plasmonic Photothermal Nanoagents. ACS Nano.

[8] Ouyang J, Xie A, Zhou J, Liu R, Wang L, Liu H, Kong N, Tao W. Minimally invasive nanomedicine: nanotechnology in photo-/ultrasound-/radiation-/magnetism-mediated therapy and imaging. Chem Soc Rev. 2022;51(12):4996–5041 10.1039/d1cs01148k 35616098

[9] Ng CW, Li JC, Pu KY. Recent Progresses in Phototherapy-Synergized Cancer Immunotherapy. Adv Funct Mater 2018, 28.

[10] Xie ZJ, Fan TJ, An J, Choi W, Duo YH, Ge YQ, Zhang B, Nie GH, Xie N, Zheng TT (2020). Emerging combination strategies with phototherapy in cancer nanomedicine. Chem Soc Rev.

[11] Wei J, Liu Y, Yu J, Chen L, Luo M, Yang L, Li P, Li S, Zhang XH (2021). Conjugated Polymers: Optical Toolbox for Bioimaging and Cancer Therapy. Small.

[12] Yang X, Ma L, Shao H, Zhou Z, Ling X, Yao M, Luo G, Scoditti S, Sicilia E, Mazzone G et al. Riboflavin-promoted in situ photoactivation of dihydroalkaloid prodrugs for cancer therapy. J Med Chem. 2022;65(23):15738–48. 10.1021/acs.jmedchem.2c01262 36410876

[13] Li J, Dai J, Zhuang Z, Meng Z, Hu JJ, Lou X, Xia F, Zhao Z, Tang BZ (2022). Combining PD-L1 blockade with immunogenic cell death induced by AIE photosensitizer to improve antitumor immunity. Biomaterials.

[14] Zheng Q, Liu X, Zheng Y, Yeung KWK, Cui Z, Liang Y, Li Z, Zhu S, Wang X, Wu S (2021). The recent progress on metal-organic frameworks for phototherapy. Chem Soc Rev.

[15] Li C, Jiang G, Yu J, Ji W, Liu L, Zhang P, Du J, Zhan C, Wang J, Tang BZ. Fluorination enhances NIR-II emission and photothermal conversion efficiency of phototheranostic agents for imaging-guided cancer therapy. Adv Mater 2022:e2208229. 10.1002/adma.202208229 36300808

[16] Feng L, Li C, Liu L, Chen X, Jiang G, Wang J, Tang BZ (2022). A Facile Structural Isomerization-Induced 3D spatial D-A Interlocked Network for Achieving NIR-II Phototheranostic Agents. Angew Chem Int Ed Engl.

[17] Yan D, Li T, Yang Y, Niu N, Wang D, Ge J, Wang L, Zhang R, Wang D, Tang BZ. A Water-Soluble AIEgen for Noninvasive diagnosis of kidney fibrosis via SWIR fluorescence and photoacoustic imaging. Adv Mater 2022:e2206643. 10.1002/adma.202206643 36222386

[18] Li S, Chen H, Liu H, Liu L, Yuan Y, Mao C, Zhang W, Zhang X, Guo W, Lee CS, Liang XJ (2020). In vivo real-time Pharmaceutical evaluations of Near-Infrared II fluorescent nanomedicine bound polyethylene glycol ligands for Tumor Photothermal ablation. ACS Nano.

[19] Zhu J, He G, Chen PH, Zhang Y, Zhang Y, Lei S, Zhang Y, Li M, Huang P, Lin J (2023). Terpyridine-Grafted Nitrogen-Terminal endowing cyanine with metal-ion-regulated Photophysical Properties for Cancer Theranostics. Res (Wash D C).

[20] Shi Z, Bai H, Wu J, Miao X, Gao J, Xu X, Liu Y, Jiang J, Yang J, Zhang J et al. Acceptor engineering produces ultrafast nonradiative decay in NIR-II Aza-BODIPY nanoparticles for efficient Osteosarcoma Photothermal Therapy via concurrent apoptosis and pyroptosis. Research. 2023;6:0169. 10.34133/research.0169 PMC10278946 37342631

[21] Liu Y, Bhattarai P, Dai Z, Chen X (2019). Photothermal therapy and photoacoustic imaging via nanotheranostics in fighting cancer. Chem Soc Rev.

[22] Xu C, Jiang Y, Han Y, Pu K, Zhang R (2021). A polymer multicellular nanoengager for synergistic NIR-II Photothermal Immunotherapy. Adv Mater.

[23] Leitao MM, de Melo-Diogo D, Alves CG, Lima-Sousa R, Correia IJ. Prototypic heptamethine cyanine incorporating nanomaterials for cancer phototheragnostic. Adv Healthc Mater. 2020;9(6):e1901665. 10.1002/adhm.201901665 31994354

[24] He W, Zhang Z, Luo Y, Kwok RTK, Zhao Z, Tang BZ (2022). Recent advances of aggregation-induced emission materials for fluorescence image-guided surgery. Biomaterials.

[25] Yuan HX, Li ZL, Zhao Q, Jia SC, Wang T, Xu L, Yuan HT, Li SL. Molecular evolution of acceptor-donor-acceptor-type conjugated oligomer nanoparticles for efficient photothermal antimicrobial therapy. Adv Funct Mater. 2023;33:2213209.

[26] Fan X, Xia Q, Zhang Y, Li Y, Feng Z, Zhou J, Qi J, Tang BZ, Qian J, Lin H (2021). Aggregation-Induced Emission (AIE) Nanoparticles-Assisted NIR-II fluorescence imaging-guided diagnosis and surgery for inflammatory bowel disease (IBD). Adv Healthc Mater.

[27] Liu S, Ou H, Li Y, Zhang H, Liu J, Lu X, Kwok RTK, Lam JWY, Ding D, Tang BZ (2020). Planar and twisted molecular structure leads to the high brightness of semiconducting polymer nanoparticles for NIR-IIa fluorescence imaging. J Am Chem Soc.

[28] Fan XX, Li YR, Feng Z, Chen GQ, Zhou J, He MB, Wu L, Li SL, Qian J, Lin H. Nanoprobes-Assisted multichannel NIR-II fluorescence imaging-guided resection and photothermal ablation of Lymph Nodes. Adv Sci 2021, 8. 10.1002/advs.202003972 PMC8097375 33977058

[29] Dai HM, Cheng ZJ, Zhang T, Wang WL, Shao JJ, Wang WJ, Zhao YX, Dong XC, Zhong LP (2022). Boron difluoride formazanate dye for high-efficiency NIR-II fluorescence imaging-guided cancer photothermal therapy. Chin Chem Lett.

[30] Deng G, Peng X, Sun Z, Zheng W, Yu J, Du L, Chen H, Gong P, Zhang P, Cai L, Tang BZ (2020). Natural-killer-cell-inspired Nanorobots with Aggregation-Induced Emission characteristics for Near-Infrared-II fluorescence-guided glioma theranostics. ACS Nano.

[31] Pang Z, Yan W, Yang J, Li Q, Guo Y, Zhou D, Jiang X (2022). Multifunctional gold nanoclusters for effective targeting, Near-Infrared fluorescence imaging, diagnosis, and treatment of Cancer Lymphatic Metastasis. ACS Nano.

[32] Su Y, Yu B, Wang S, Cong H, Shen Y (2021). NIR-II bioimaging of small organic molecule. Biomaterials.

[33] Dai H, Wang X, Shao J, Wang W, Mou X, Dong X (2021). NIR-II Organic Nanotheranostics for Precision Oncotherapy. Small.

[34] Shao W, Zhao F, Xue J, Huang L (2023). NIR-II absorbing organic nanoagents for photoacoustic imaging and photothermal therapy. BMEMat.

[35] Lin G, Manghnani PN, Mao D, Teh C, Li Y, Zhao Z, Liu B, Tang BZ (2017). Robust Red Organic Nanoparticles for in vivo fluorescence imaging of Cancer Cell Progression in Xenografted zebrafish. Adv Funct Mater.

[36] Tang Y, Li Y, Lu X, Hu X, Zhao H, Hu W, Lu F, Fan Q, Huang W (2019). Bio-Erasable Intermolecular Donor–Acceptor Interaction of Organic Semiconducting Nanoprobes for Activatable NIR-II fluorescence imaging. Adv Funct Mater.

[37] Yin C, Tai XY, Li XZ, Tan JH, Lee CS, Sun PF, Fan QL, Huang W. Side chain engineering of semiconducting polymers for improved NIR-II fluorescence imaging and photothermal therapy. Chem Eng J 2022, 428.

[38] Xu C, Pu K (2021). Second near-infrared photothermal materials for combinational nanotheranostics. Chem Soc Rev.

[39] Ma G, Liu Z, Zhu C, Chen H, Kwok RTK, Zhang P, Tang BZ, Cai L, Gong P (2022). H(2) O(2) -Responsive NIR-II AIE Nanobomb for Carbon Monoxide Boosting Low-Temperature Photothermal Therapy. Angew Chem Int Ed Engl.

[40] Liu S, Li Y, Kwok RTK, Lam JWY, Tang BZ (2020). Structural and process controls of AIEgens for NIR-II theranostics. Chem Sci.

[41] Li N, Gao Y, Li B, Gao D, Geng H, Li S, Xing C (2022). Remote manipulation of ROS-Sensitive Calcium Channel using Near-Infrared-Responsive Conjugated Oligomer Nanoparticles for enhanced tumor therapy in vivo. Nano Lett.

[42] Li X, Fang F, Sun B, Yin C, Tan J, Wan Y, Zhang J, Sun P, Fan Q, Wang P (2021). Near-infrared small molecule coupled with rigidness and flexibility for high-performance multimodal imaging-guided photodynamic and photothermal synergistic therapy. Nanoscale Horiz.

[43] Yu YJ, Tang DS, Liu CY, Zhang Q, Tang L, Lu YF, Xiao HH. Biodegradable polymer with effective Near-Infrared-II absorption as a Photothermal Agent for Deep Tumor Therapy. Adv Mater 2022, 34. 10.1002/adma.202105976 34695252

[44] Li S, Deng Q, Zhang Y, Li X, Wen G, Cui X, Wan Y, Huang Y, Chen J, Liu Z (2020). Rational design of Conjugated Small Molecules for Superior Photothermal Theranostics in the NIR-II Biowindow. Adv Mater.

[45] Luo T, Jiang M, Cheng Z, Lin Y, Chen Y, Zhang Z, Zhou J, Zhou W, Yu XF, Li S (2023). Biodegradable FePS(3) nanoplatform for efficient treatment of osteosarcoma by combination of gene and NIR-II photothermal therapy. J Nanobiotechnol.

[46] Yang G, Phua SZF, Bindra AK, Zhao Y (2019). Degradability and clearance of Inorganic Nanoparticles for Biomedical Applications. Adv Mater.

[47] Lenders V, Koutsoumpou X, Phan P, Soenen SJ, Allegaert K, de Vleeschouwer S, Toelen J, Zhao Z, Manshian BB (2023). Modulation of engineered nanomaterial interactions with organ barriers for enhanced drug transport. Chem Soc Rev.

[48] Mahmoud K, Swidan S, El-Nabarawi M, Teaima M. Lipid based nanoparticles as a novel treatment modality for hepatocellular carcinoma: a comprehensive review on targeting and recent advances. J Nanobiotechnol 2022, 20. 10.1186/s12951-022-01309-9 PMC8898455 35248080

[49] Izci M, Maksoudian C, Manshian BB, Soenen SJ (2021). The use of alternative strategies for enhanced nanoparticle delivery to solid tumors. Chem Rev.

[50] Li X, Liu L, Li S, Wan Y, Chen JX, Tian S, Huang Z, Xiao YF, Cui X, Xiang C (2019). Biodegradable pi-conjugated oligomer nanoparticles with high Photothermal Conversion Efficiency for Cancer Theranostics. ACS Nano.

[51] Lee KW, Gao Y, Wei WC, Tan JH, Wan Y, Feng Z, Zhang Y, Liu Y, Zheng X, Cao C (2023). Anti-quenching NIR-II J-Aggregates of Benzo[c]thiophene fluorophore for highly efficient bioimaging and phototheranostics. Adv Mater.

[52] Sun C, Li B, Zhao M, Wang S, Lei Z, Lu L, Zhang H, Feng L, Dou C, Yin D (2019). J-Aggregates of cyanine dye for NIR-II in vivo dynamic vascular imaging beyond 1500 nm. J Am Chem Soc.

