# Supplementary material for: Selenium-integrated conjugated oligomer nanoparticles with high photothermal conversion efficiency for NIR-II imaging-guided cancer phototheranostics in vivo
Source: J Nanobiotechnology. 2023 Sep 4;21:314. doi: 10.1186/s12951-023-02080-1 (PMC10476403; doi:10.1186/s12951-023-02080-1)
Supplement: Supplementary file 1 — Additional file 1 (1) Materials and methods 1.1. The encapsulating efficiency of the NPs. (2) Results Figure S1: Synthetic route of TPSe; Figure S2: 1 H NMR spectra of TPSe; Figure S3: 13 C NMR spectra of TPSe; Figure S4: High-resolution mass spectrometry of TPSe; Figure S5: UV-VIS-NIR absorption spectrum of TPSe; Figure S6: HOMO and LUMO of TPSe; Figure S7: Absorption spectra of TPSe before and after laser irradiation; Figure S8: Absorption spectra of ICG before and after laser irradiation; Figure S9: Size distribution of TPSe before and after laser irradiation; Figure S10: Zeta potential of TPSe NPs before and after irradiation; Figure S11: Detection of in vitro ROS generation; Figure S12: Toxicity of TPSe NPs in 4T1, MGC-803, HepG2 cell; Figure S13: Fluorescence image of major organs after injection of TPSe NPs; Table S1: The comparison of TPSe NPs with reported nanomaterials. [file 12951_2023_2080_MOESM1_ESM.docx]

Supporting Information

**Selenium-Integrated Conjugated Oligomer Nanoparticles with High Photothermal Conversion Efficiency for NIR-II Imaging-Guided Cancer Phototheranostics *In Vivo***

Lele Yang, Yijian Gao, Jinchao Wei, Zehua Cheng, Sijia Wu, Liang Zou, Shengliang Li* and Peng Li*

**1. Materials and methods**

**1.1. The encapsulating efficiency of the NPs**

A proper number of materials was weighed and prepared into THF solution (1.0 mg/mL). Then, it was diluted into THF solutions with different concentrations (50, 25, 12.5, 6.25, 0 μg/mL). The absorbance at the highest peak (830 nm) was determined to be A1-A5 (1.28, 0.667, 0.341, 0.175, 0) respectively. Fitting the curve of absorbance and concentration: y=0.025504x+0.0144, R2=0.99942. Concentrate the prepared nanoparticles by ultrafiltration, take appropriate NPs solution, and dissolve it in 2 mL of THF to depolymerize it into molecular state (dilution multiple is n), measure the absorbance Ax after fully mixing. The concentration of concentrated NPs stock solution (1):

(1)

$$C= (Ax-0.0144)/0.025504 *n$$

Record the mass M0 of TPSe before preparing NPs, freeze-dry the prepared NPs aqueous solution through 300 kDa filter membrane, and weigh the mass M1 of solid powder. The solid powder was dissolved in ultrapure water with an appropriate volume V, and the solution concentration was determined to be C according to the above method. Then the encapsulation efficiency is (2):

(2)

$$C*V/M0$$

And the drug loading rate is (3):

(3)

$$C*V/M1$$

**2. Results**


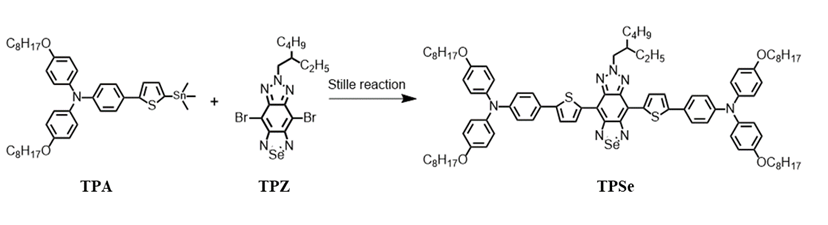


**Figure S1.** Synthetic route of compound TPSe.


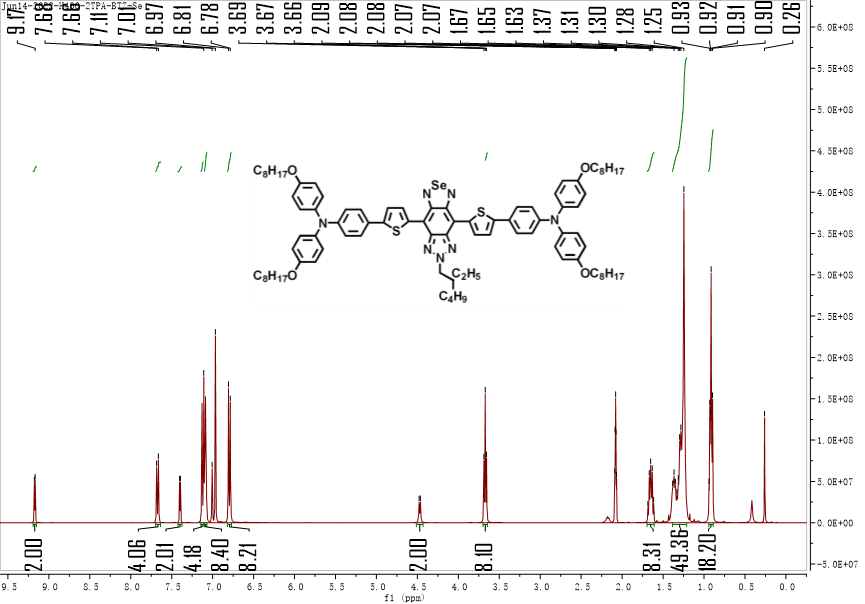


**Figure S2.** ^1^H NMR spectra of compound TPSe (400 MHz, CD_2_Cl_2_).


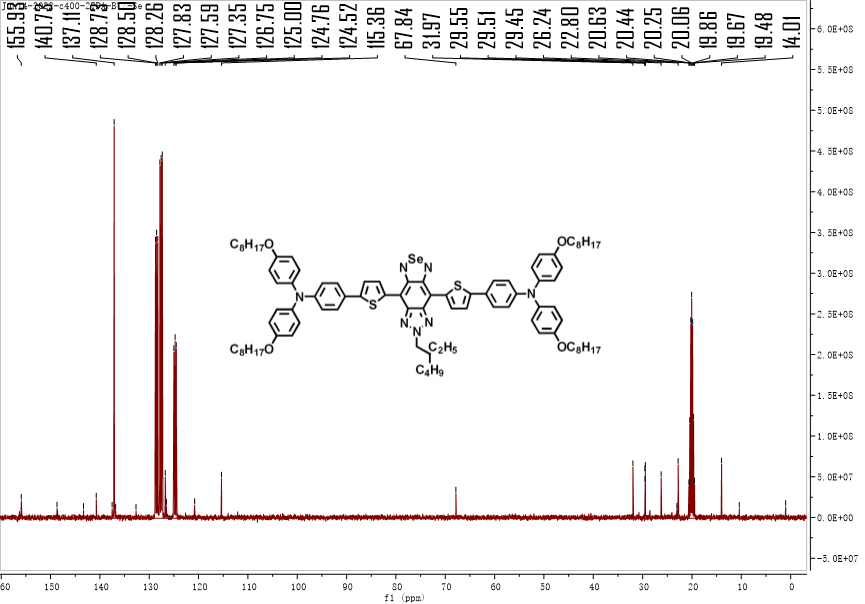


**Figure S3.** ^13^C NMR spectra of compound TPSe (101 MHz, CD_2_Cl_2_).


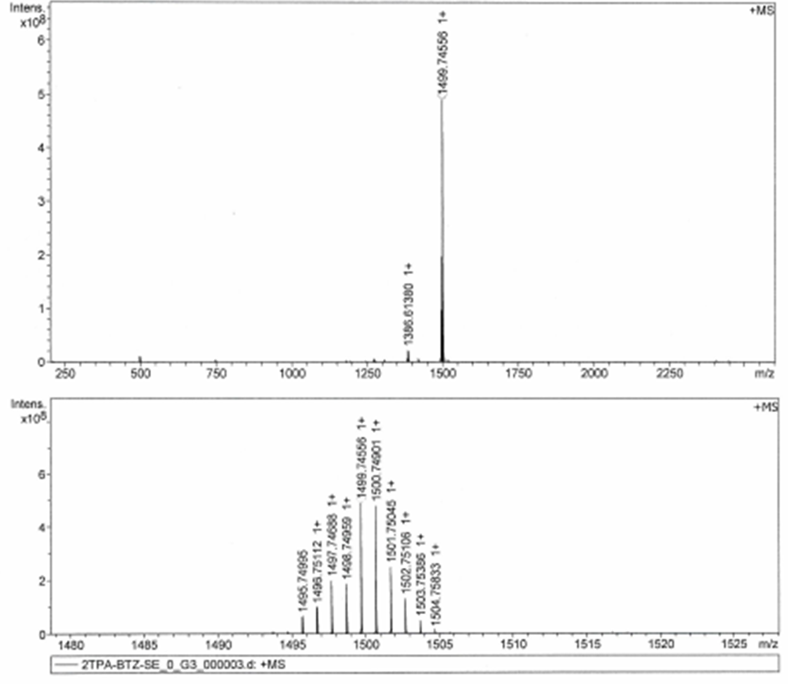


**Figure S4.** Matrix assisted laser desorption/ionization time-of-flight (MALDI-TOF) mass spectrum of compound TPSe.


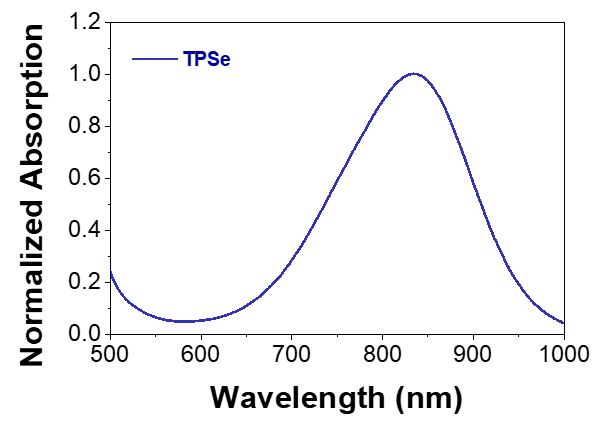


**Figure S5.** Normalized absorption spectrum of TPSe (THF).


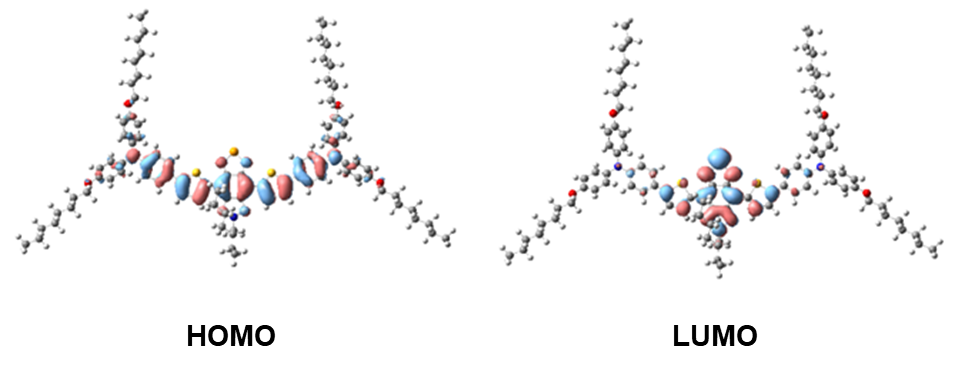


**Figure S6.** The highest occupied molecular orbital (HOMO) and the lowest unoccupied molecular orbital (LUMO) of TPSe.


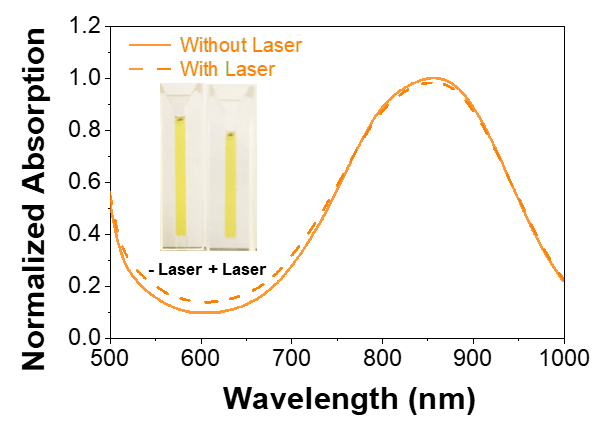


**Figure S7.** Absorption spectra of TPSe NPs irradiated by 808 nm laser at 1 W cm^−2^ for 1 h before and after. Insets are corresponding photographs of TPSe NPs before and after laser irradiation.


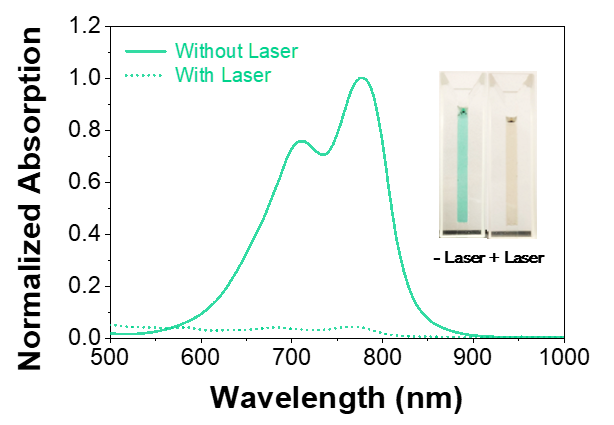


**Figure S8.** Absorption spectra of ICG solutions irradiated by 808 nm laser at 1 W cm^−2^ for 1 h before and after. Insets are corresponding photographs of ICG solutions before and after laser irradiation.


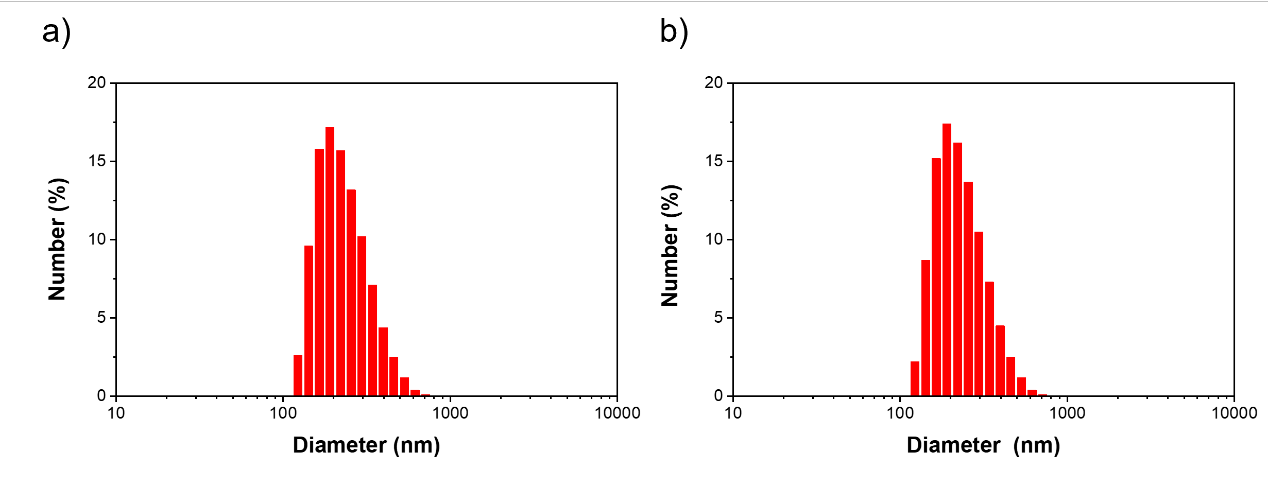


**Figure S9.** DLS profile of TPSe NPs before and after laser irradiation.


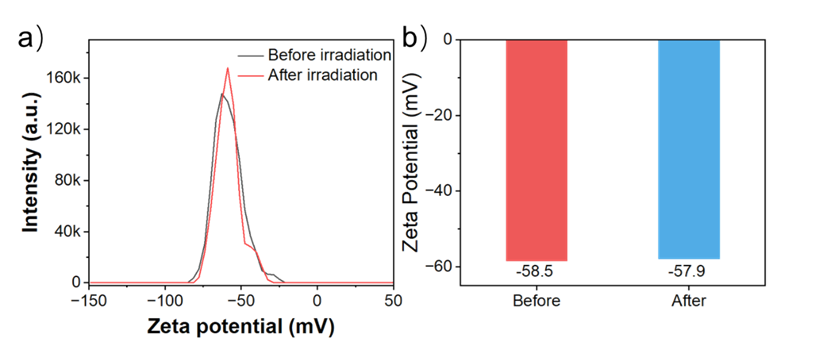


**Figure S10.** Zeta potential of TPSe NPs before and after irradiation (808 nm laser, 1.0 W cm^-2^, 10 min).


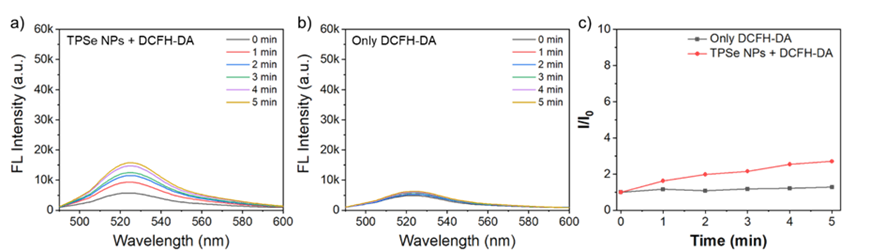


**Figure S11.** a, b) Detection of ROS generated with or without TPSe NPs using DCFH-DA sensor (1.0 W cm^−2^ at 808 nm) as control. c) Comparison of fluorescence intensity changes of DCFH-DA with or without TPSe NPs.


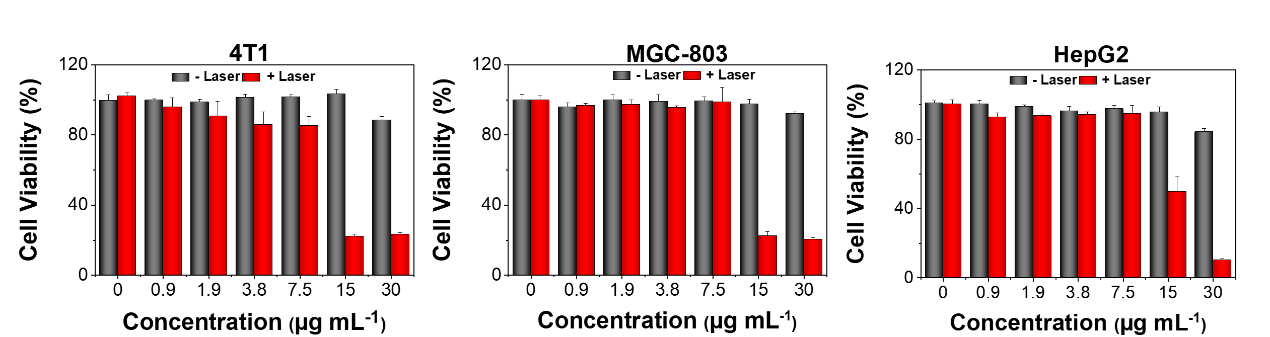


**Figure S12.** *In vitro* survival rate of TPSe NPs-treated cancer cell lines in the absence or presence of 808 nm laser irradiation at 0.5 W cm^−2^ for 5 min.


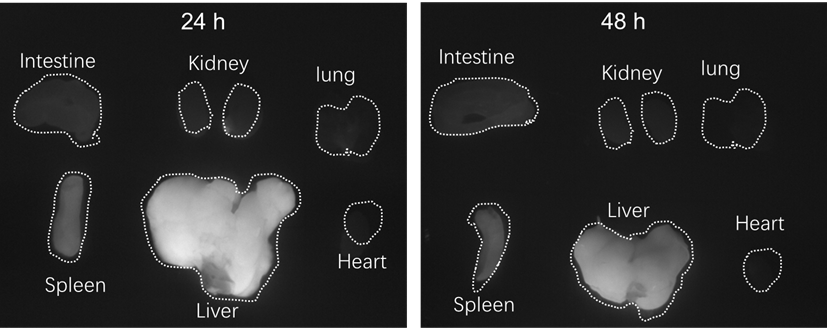


**Figure S13.** NIR-II FL imaging of major organs of the mouse after 24 h and 48 h post-injection of TPSe NPs (0.5 mg mL^-1^).

**Table S1**. The comparison of TPSe NPs with reported nanomaterials.

| Nanoparticles | Advantage | Size (nm) | Photothermal conversion efficiency (η) | Laser wavelength (nm) | Laser irradiation time (min) | Cancer cells | Temperature | Ref. |
| --- | --- | --- | --- | --- | --- | --- | --- | --- |
| TPSe-based NPs | Fluorescence imaging | 200 | 60.29% | 808 nm (0.5 W/cm^2^) | 5 | *In vitro*: HCT116, A549, 4T1, MGC-803, HepG2 cells; *In vivo*: HCT116 cells | 57.9 ℃ | The preset study |
| HA-conjugated hydroxychloroquine NPs | Chemo-photothermal therapy | 251.9 | NA | 808 nm (1 W/cm^2^) | 5 | *In vitro*: DLD-1 and HCT116 cells; *In vivo*: DLD-1 cells | 30.7 ℃ | [1] |
| FA-CS-R-Pd NCs | Folic acid conjugated chitosan | 129.7 ± 1.01 | 15.14 ± 0.65% | 808 nm (1 W/cm^2^) | 5 | *In vitro*: HEK-293 and MDA-MB cells | 51.7 °C | [2] |
| Fe3O4/TMB-PEG-RGD (FTRNPs) | Dual-targeting nanozyme | 16.41 ± 3.19 | 50.90% | 808 nm (2 W/cm^2^) | 10 | *In vitro* and *in vivo*: 4T1 cells | 61.7 °C | [3] |
| APP NPs | Chemo-photothermal therapy | 105 | 30% | 660 nm (0.8 W/cm^2^) | 5 | *In vitro* and *in vivo*: 4T1 cells | 57 °C | [4] |
| IT-TQF NPs | Fluorescence imaging | 12 | 47% | 808 nm (1 W/cm^2^) | 10 | *In vivo*: 143B cells | 57.5 °C | [5] |
| trans-CP NPs | PA imaging | 70 | 61.40% | 808 nm (0.75 W/cm^2^) | 5 | *In vitro*: A549, H446 cells; *In vivo*: A549 cells | 65 °C | [6] |

1. Wang SQ, Zhou L, Tian HL, Li BW, Su M, Li Q, Nice EC, Huang CH, Shao JC, He T: Site-specific nanomodulator capable of modulation apoptosis for enhanced colorectal cancer chemo-photothermal therapy. Journal of Nanobiotechnology 2023, 21.

2. Sathiyaseelan A, Saravanakumar K, Manivasagan P, Jeong MS, Jang ES, Wang MH: Folic acid conjugated chitosan encapsulated palladium nanoclusters for NIR triggered photothermal breast cancer treatment. Carbohydrate Polymers 2022, 280:119021.

3. Chen CY, Chen YW, Zhang LL, Wang XH, Tang QS, Luo Y, Wang Y, Ma C, Liang XL: Dual-targeting nanozyme for tumor activatable photo-chemodynamic theranostics. Journal of Nanobiotechnology 2022, 20.

4. Zhang W, Zhang C, Yang C, Wang X, Liu W, Yang M, Cao Y, Ran H: Photochemically-driven highly efficient intracellular delivery and light/hypoxia programmable triggered cancer photo-chemotherapy. Journal of Nanobiotechnology 2023, 21:11.

5. Lou HY, Ji AY, Qu CR, Liu HG, Jiang L, Chen H, Cheng Z: A Small-Molecule Based Organic Nanoparticle for Photothermal Therapy and Near-Infrared-IIb Imaging. ACS Applied Materials & Interfaces 2022, 14:35454-35465.

6. Gao Y, Li Q, Li C, Gao L, Chen H, Liu T, Huang Y, Liu Z, Li S: Terselenophene Regioisomer Conjugated Polymer Materials for High-Performance Cancer Phototheranostics. ACS Applied Materials & Interfaces 2020, 12:55605-55613.
